# Bryophytes Harbor Cultivable Actinobacteria With Plant Growth Promoting Potential

**DOI:** 10.3389/fmicb.2020.563047

**Published:** 2020-09-29

**Authors:** Chadabhorn Insuk, Nattakorn Kuncharoen, Naowarat Cheeptham, Somboon Tanasupawat, Wasu Pathom-aree

**Affiliations:** ^1^Master of Science Program in Applied Microbiology, Faculty of Science, Chiang Mai University, Chiang Mai, Thailand; ^2^Department of Biology, Faculty of Science, Chiang Mai University, Chiang Mai, Thailand; ^3^Department of Biochemistry and Microbiology, Faculty of Pharmaceutical Sciences, Chulalongkorn University, Bangkok, Thailand; ^4^Department of Biological Sciences, Faculty of Science, Thompson Rivers University, Kamloops, BC, Canada; ^5^Research Center of Microbial Diversity and Sustainable Utilization, Faculty of Science, Chiang Mai University, Chiang Mai, Thailand

**Keywords:** actinobacteria, bryophytes, *Physcomitrium sphaericum*, plant-microbes interaction, plant-growth promoting bacteria, genome mining, draft genome, whole genome sequencing

## Abstract

This study was designed to investigate the cultivable actinobacteria associated with bryophytes and their plant growth promoting ability. Thirteen actinobacteria were isolated and tested for their ability to promote growth of plant *in vitro* and *in planta*. All isolates were able to produce IAA and siderophores. Six isolates were identified as members of the genus *Micromonospora*. Five isolates belonged to the genus *Streptomyces* and one each of *Microbispora* and *Mycobacterium*. *Micromonospora* sp. CMU55-4 was inoculated to rare moss [*Physcomitrium sphaericum* (C. Ludw.) Fürnr.] and could increase the amount of carotenoid, fresh weight, and dry weight of this moss. In addition, this strain promoted capsule production, and rescued *P. sphaericum*’s gametophytes during acclimatization to land. Strain CMU55-4 was identified as *Micromonospora chalcea* based on whole genome sequence analysis. Its plant growth promoting potential was further characterized through genome mining. The draft genome size was 6.6 Mb (73% GC). The genome contained 5,933 coding sequences. Functional annotation predicted encoded genes essential for siderophore production, phosphate solubilization that enable bacteria to survive under nutrient limited environment. Glycine-betaine accumulation and trehalose biosynthesis also aid plants under drought stress. *M. chalcea* CMU55-4 also exhibited genes for various carbohydrate metabolic pathways indicating those for efficient utilization of carbohydrates inside plant cells. Additionally, predictive genes for heat shock proteins, cold shock proteins, and oxidative stress such as glutathione biosynthesis were identified. In conclusion, our results demonstrate that bryophytes harbor plant growth promoting actinobacteria. A representative isolate, *M. chalcea* CMU55-4 promotes the growth of *P. sphaericum* moss and contains protein coding sequences related to plant growth promoting activities in its genome.

## Introduction

Plant microbiomes are involved in the well-being of their host health by inhibiting the growth of plant pathogens and help plant to tolerate stress conditions (Gabriele, 2016). Plant growth-promoting rhizobacteria (PGPR), which live around root area of plants, can be beneficial to plants by mediating plant growth through diverse biochemical mechanisms such as biosynthesis of indole-3-acetic acid (IAA), 1-aminocyclopropane-1-carboxylic acid (ACC) deaminase, siderophore production, and phosphate solubilization. PGPR which are abundant in soil and rhizosphere can penetrate into root endosphere through cellular disjunction during lateral root emergence (Bulgarelli et al., 2013) to colonize inside plants. *Actinobacteria*, a phylum of Gram-positive bacteria that contained high amount of G+C in their DNA (>55 mol% of genomic DNA) are interesting due to their prolific metabolic capabilities (Yadav et al., 2018). Plant growth promoting actinobacteria (PGPA) are abundant in soil and can enhance nutrients availability, administer plant metabolism and reduce environmental stress in plants (Hamedi and Mohammadipanah, 2014). Bryophytes are group of plants that grow under specific climate conditions and their microbiomes are still not well studied. They are the oldest known land plants that lack true leaves, stem or true vascular system and considered as a remarkable reservoir of novel active compounds, natural products and antibiotics (Chandra, 2014). Actinobacteria associated with bryophytes could have some specific evolutionary adaptations to their host plants and may have potential of bioactive compounds production that need to be investigated. One of the easiest ways for bryophytes propagation is using tissue culture technique. However, the obtained *in vitro* plantlets are usually unable to compete with other native soil microbes when transfer to soil. In addition, micropropagated plants are struggled to cope with environmental conditions in the field which results in the morphological and anatomical changes of plantlets (Chandra and Bandopadhyay, 2010). The objectives of this study are

1. To investigate the cultivable actinobacteria associated with some bryophytes;
2. To study the plant growth promoting ability of actinobacteria from bryophytes.

One strain, *Micromonospora chalcea* CMU55-4 shows promising potential to promote the growth of tested bryophyte during acclimatization period in laboratory before transfer to the forest. Its taxonomic characterization and whole genome analysis are also reported.

## Materials and Methods

### Sample Collection

Bryophyte samples were collected from Doi Inthanon National Park, Chiang Mai, Thailand. Various keys and checklists were used for identification such as Hattori et al. (1977), Eddy (1988, 1996), Zander (1993), and Li et al. (2007). Actinobacteria were isolated from five species of bryophytes, including mosses and liverwort. Four high altitude moss species were *Bryum apiculatum* Schwägr. (CMU15), *Syntrichia gemmascens* (P.C. Chen) R.H. Zander (CMU51), *Campylopus involutus* (Müll. Hal.) A. Jaeger (CMU55), and *Plagiomnium maximoviczii* (Lindb.) T.J. Kop (CMU13). Liverwort was *Frullania nepalensis* (Spreng.) Lehm. and Lindenb. (CMU12). The remaining bryophyte samples from isolation were kept at Chiang Mai University Herbarium (Herbarium code: CMUB).

### Selective Isolation

Each bryophyte sample (1 g fresh weight) was surface sterilized (70% ethanol 1 min, 3% sodium hypochlorite 10 min, 3 times sterile distilled water). Sterile samples were crushed in sterile mortars, diluted with sterile distilled water up to 10^–3^ and plated on starch casein agar (Küster and Williams, 1964), humic acid vitamin B agar (Hayakawa and Nonomura, 1987), R2A agar and water proline agar (1% proline). All media were supplemented with 25 μg/ml nystatin and 10 μg/ml cycloheximide. Plates were incubated for 30 days at 25°C. All colonies appeared on agar were picked and purified on ISP2 agar (Shirling and Gottlieb, 1966). The effectiveness of the surface sterilization method was evaluated by spread the final rinse water of each bryophyte sample on ISP2 agar.

### Plant Growth Promoting Activity

IAA production was determined following the methods of Glickman and Dessaux (1995). In brief, actinobacteria were grown in ISP2 broth with and without 2 mg/ml L-tryptophan for 7 days in the dark. After that, the supernatant was mixed with Salkowski’s reagent (1 ml of 0.5 M FeCl_3_ in 50 ml 35% HClO_4_), stored at room temperature for 30 min and measured the optical density at 530 nm. IAA production is also confirmed by HPLC (Fluorescent detector) with a Restek Ultra C18, 5 μm (150 × 4.6 mm) using 0.1 M acetic acid as mobile phase A and 0.1 M acetic acid in methanol as mobile phase B (1 ml/min flow rate). Standard methods were used to determine siderophore production qualitatively and quantitatively. Siderophore production was first screened on CAS agar (Schwyn and Neilands, 1987) using King’s B agar as a basal medium (King et al., 1954). The appearance of a yellow to orange zone around the actinobacterial colony after 7 days incubation in the dark was an indication of siderophore production. Quantitative determination of siderophore was carried out in liquid medium. *Actinobacteria* were grown in King’s B broth for 7 days. The culture supernatant was mixed with ferric perchlorate solution for hydroxamate siderophore (Atkin et al., 1970), and 0.5 M HCl, nitrite-molybdate reagent and 1 M NaOH for catecholate siderophore (Arnow, 1937). Phosphate solubilization was determined from clear zone formation on Pikovskaya (PVK) agar supplemented with 0.5% (w/v) tricalcium phosphate after 7 days incubation (Nautiyal, 1999). Drought tolerant ability of actinobacteria was also investigated using sorbitol as an osmotic adjustment in water agar medium (Hallsworth et al., 1998).

### Taxonomic Characterization

DNA extraction was performed following the method of Kudo et al. (1998). Genomic DNA was used as the template for polymerase chain reaction (PCR) following the method of Lee et al. (2014). 27F (5′ AGAGTTTGATCMTGGCTCAG 3′) and 1492R (5′ TACGGYTACCTTGTTACGACTT 3′) primers were used to amplify 16S rRNA gene. The PCR amplicons were purified by GF-1 AmbiClean Kit (Vivantis^®^) following the manufacturer instruction. The 16S rRNA sequencing was performed by commercial service at *First BASE* Laboratories Sdn Bhd, Malaysia. Identification of all actinobacterial isolates was achieved by BLAST analysis of 16S rRNA gene sequences using EzBiocloud database^1^. Neighbor-joining phylogenetic tree was constructed using BioEdit Sequence Alignment Editor version 7.2 and MEGA7 (Kumar et al., 2016). Tree topology was evaluated using the bootstrap resampling method at 1000 bootstrap. Strain CMU55-4 was also identified based on whole genome sequence analysis. The ANI value was calculated and compared in JSpeciesWS (Ritcher et al., 2016), web server tool, using ANI-Blast (ANIb) and ANI-MUMmer (ANIm) algorithms (Ritcher and Rosselló-Móra, 2009) within the web service. The Genome-to-Genome Distance Calculator (GGDC 2.1) with the BLAST + method (Meier-Kolthoff et al., 2013) was used to evaluate the digital DNA-DNA hybridisation (dDDH).

### Growth Promotion on *Physcomitrium sphaericum* (C. Ludw.) Fürnr.

*Physcomitrium sphaericum* (C. Ludw.) Fürnr. was used as a moss model to determine beneficial effects of selected actinobacteria on bryophytes during transplantation. *P. sphaericum* is a rare moss species in Thailand with the risk from extinction. The re-introduction of this moss back to nature is badly needed. Spores of *P. sphaericum* were picked from healthy plants from the nature under stereo microscope using sterile forceps and placed into Hoagland agar (Hoagland and Arnon, 1950) and incubated for 3 months at 24°C, 35,000–40,000 lux intensity to let spore germinate and form protonema. Then, the moss protonema was transferred to Hoagland agar and incubated in the same condition for 3 months. The moss plantlets were transferred from agar media to soil. Strain CMU55-4 was chosen to be introduced to moss due to its high IAA production *in vitro* and fast growth within 3 days which is an important trait of PGPA to compete with other soil microbes. Strain CMU55-4 was grown in ISP2 broth for 7 days, 25°C, 150 rpm. Culture broth was centrifuged to collect cells. Cells were washed and resuspended in sterile distilled water and adjusted the concentration to OD_600_ = 1, which is equivalent to 10^6^ cells/ml. A suspension of strain CMU55-4 (1 ml) was dropped into the autoclaved soil around the plants. Sterile distilled water was used as a control solution. The plants were maintained in the incubator at 24°C, 35,000–40,000 lux for 1 month. Chlorophyll and carotenoid contents were determined from fresh samples using standard method (Chappelle et al., 1992). Dry weight was obtained from plants that were dried in 60°C oven for 7 days.

### Genome Mining for Plant Growth Promoting Potential

Whole genome sequencing of *Micromonospora* sp. CMU55-4 was carried out by an Illumina Miseq platform (Illumina, Inc., San Diego, US-CA) using 2 × 250 bp paired-end reads. Raw reads quality was checked using FASTQC software (Andrews, 2010). Adaptors and poor-quality reads were removed using Trim Galore (Krueger, 2015), and the filtered reads were used as an input for Unicycler (Wick et al., 2017), genome assembly program. Annotation of assembled genome was done using Prokka Version 1.13 (Seemann, 2014), and NCBI Prokaryotic Genome Annotation Pipeline (PGAP). The draft genome sequence of strain CMU55-4 was mined using RAST annotation server (Aziz et al., 2008) and analyzed through SEED viewer (Overbeek et al., 2014) for genes responsible for plant growth promoting properties.

### GenBank Accession Number

The accession number of the draft genome sequence is JAAOLH000000000.

### Chemotaxonomic, Cultural, and Phenotypic Characterization of *Micromonospora* sp. CMU55-4

The type strain *Micromonospora chalcea* DSM 43026^*T*^ was purchased from Deutsche Sammlung von Mikroorganismen und Zellkulturen (DSM) culture collection. *Micromonospora terminaliae* TMS7^*T*^ was kindly given by Dr. Onuma Kaewkla of Department of Biology, Faculty of Science, Mahasarakham University. Cultural characteristics were determined after 2 weeks at 30°C on ISP1-7 media (Shirling and Gottlieb, 1966), Nutrient agar (Difco), Tryptic Soy agar (Difco), Potato Dextrose agar (Difco), Modified Bennett agar (Jones, 1949), and ATCC172 agar. The ISCC-NBS color chart (Kelly, 1964) was used to determine the color of mycelia. pH range for growth (4.0–12.0 at intervals of 1.0 pH unit), tolerant to NaCl concentration (0–9% at 1% intervals) and temperature range for growth (4, 20, 25, 30, 37, 40, 45, 50, 55°C) were determined using ISP2 agar as a basal medium. H_2_S production, coagulation and peptonization, gelatin liquefaction, starch hydrolysis and nitrate reduction were determined on ISP6 agar, 10% skim milk (Difco), glucose-peptone-gelatin medium (2.0% glucose, 0.5% peptone, 20% gelatin pH 7.0), ISP4 agar and ISP8 broth (0.5% peptone, 0.3% beef extract, 0.1% KNO_3_, pH 7.0). The production of enzymes was determined using API ZYM kit (bioMérieux). The utilization of carbohydrates as sole carbon sources was obtained using ISP 9 (Nihon Pharmaceutical) as the basal medium supplemented with 1% (w/v) of each carbon source. Spore morphology of 21-day-old culture on ISP2 agar was observed by scanning electron microscope (JSM-IT500HR; JEOL). For chemotaxonomic study, biomass of strain CMU55-4 was obtained from culture grown in ISP2 broth for 7 days at 30°C (160 rpm) and freeze dried. The analysis of whole cell reducing sugar and isomer of diaminopimelic acid on cell wall (A_2_pm) was determined on thin-layer-chromatography (TLC) following the method of Staneck and Robert (1974). Polar lipids profiles were extracted and identified using 2-dimensional TLC according to the method of Minnikin et al. (1984). Menaquinones were extracted according to the method of Collins et al. (1977) and were analyzed by UPLC (Aligent Technology 1290 Infinity II model: 67116B 1290MCT UV detector) with a μBondapak C18 column (Waters).

### Statistical Analysis

Statistical analysis was performed by SPSS statistics 17.0 program.

## Results

### Selective Isolation

A total of 13 actinobacteria were obtained from all four selective media. The highest number of isolates was recovered on water proline agar (6 isolates, 46.15%) followed by humic acid vitamin B agar (4 isolates, 30.76%), starch casein agar (2 isolates, 15.38%) and R2A gar (1 isolate, 7.69%) (Table 2). Most isolates were from *C. involutus* (38%). However, no bacteria were obtained from liverwort.

### Plant Growth Promoting Activity

All isolates produced indole-3 acetic acid and siderophores at varying amount (Table 1). Isolates CMU51-1 and CMU55-4 produced the highest amount of IAA without L-tryptophan supplement (2.73 μg/ml) and in 2 mg/ml of L-tryptophan (11.35 μg/ml), respectively. For siderophores production, isolates CMU51-1 produced the highest amount of both hydroxamate type (992.50 ± 50.76 μMole/l) and catecholate type siderophores (484.47 ± 27.91 μMole/l) (Table 1). For drought tolerant ability, no growth was observed below a_*w*_ 0.957 for all isolates. None of the obtained isolates could solubilize tricalcium phosphate on PVK agar.

**TABLE 1:** IAA and siderophore production.

**Isolate**	**IAA (μg/ml)**		**Siderophore production**			
	**No L-tryptophan**	**2 mg/ml L-Tryptophan**	**Halozone diameter (cm)**	**Halozone color**	**Hydroxamate (μMole/l)**	**Catecholate (μMole/l)**
CMU15-1	0.70 ± 0.05^*ab*^	6.55 ± 1.43^*bc*^	0.93 ± 0.39^*a*^	Clear	60.00 ± 2.50^*a*^	20.00 ± 3.80^*a*^
CMU15-2	2.12 ± 0.15^*cd*^	4.70 ± 0.61^*abc*^	1.11 ± 0.35^*a*^	Clear	27.50 ± 0.00^*a*^	4.21 ± 0.00^*a*^
CMU15-3	1.41 ± 0.27^*abcd*^	6.62 ± 0.67^*c*^	0.95 ± 0.33^*a*^	Yellow	50.00 ± 2.50^*a*^	25.79 ± 3.16^*a*^
CMU15-4	0.68 ± 0.31^*ab*^	1.50 ± 0.42^*a*^	0.88 ± 0.24^*a*^	Yellow	110.00 ± 10.61^*a*^	5.00 ± 0.74^*a*^
CMU51-1	2.73 ± 1.23^*d*^	3.33 ± 0.07^*ab*^	1.09 ± 0.24^*a*^	Yellow	992.50 ± 50.76^*d*^	484.47 ± 27.91^*e*^
CMU51-2	2.71 ± 0.71^*d*^	3.42 ± 0.11^*abc*^	1.2 ± 0.16^*a*^	Yellow	206.25 ± 12.37^*a*^	77.11 ± 1.12^*bc*^
CMU51-4	1.82 ± 0.36^*bcd*^	3.45 ± 0.64^*abc*^	1.17 ± 0.43^*a*^	Yellow	121.25 ± 1.77^*a*^	98.68 ± 7.07^*c*^
CMU51-5	2.27 ± 0.37^*cd*^	3.53 ± 0.41^*abc*^	1.62 ± 0.18^*a*^	Yellow	610.00 ± 17.68^*bc*^	40.53 ± 7.37^*ab*^
CMU55-1	0.61 ± 0.05^*ab*^	3.5 ± 0.28^*abc*^	1.36 ± 0.16^*a*^	Yellow	478.75 ± 65.41^*b*^	11.05 ± 2.41^*a*^
CMU55-2	0.20 ± 0.07^*a*^	3.7 ± 0.66^*abc*^	1.48 ± 0.31^*a*^	Clear	42.50 ± 2.50^*a*^	10.35 ± 1.85^*a*^
CMU55-3	1.45 ± 0.32^*abcd*^	4.06 ± 0.66^*abc*^	1.27 ± 0.22^*a*^	Yellow	808.75 ± 61.87^*cd*^	289.21 ± 42.80^*d*^
CMU55-4	1.20 ± 0.05^*abc*^	11.35 ± 3.34^*d*^	0.97 ± 0.18^*a*^	Brownish yellow	54.17 ± 8.78^*a*^	10.18 ± 3.08^*a*^
CMU55-5	1.50 ± 0^*abcd*^	5.00 ± 0.08^*bc*^	1.66 ± 0.22^*a*^	Orange yellow	35.00 ± 4.33^*a*^	1.05 ± 0.74^*a*^

*^*a*–*d*^ indicated significant difference in statistical analysis tested by SPSS One-Way ANOVA and Tukey HSD test (*p* < 0.05), *n* = 3.*

### Taxonomic Characterization

**FIGURE 1:**
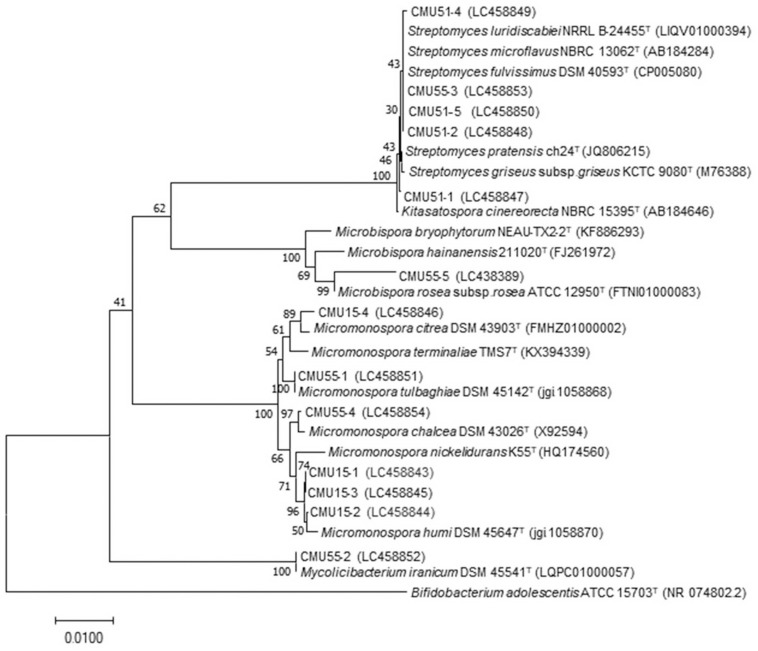
Phylogenetic tree based on neighbor-joining analysis of 16S rRNA gene sequences from bryophytes associated actinobacteria and their nearest neighbors. The numbers at branch nodes indicate bootstrap percentages derived from 1,000 replications. Bar, 0.01 substitutions per nucleotide position.

Comparison of 16S rRNA gene sequence similarity of the obtained actinobacteria in EzBiocloud database, assigned them as members of the following genera: *Micromonospora*, *Streptomyces*, *Mycolicibacterium* and *Microbispora* (Table 2). The majority of isolates were *Micromonospora* (46%) followed by *Streptomyces* (38%). The 16S rRNA gene similarity values were ranged from 96.31 to 100%. Phylogenetic analysis confirmed the assignment of these actinobacteria at genus level based on BLAST results (Figure 1). Strain CMU55-5 is closely related to *Microbispora rosea* subsp. *rosea* ATCC 12950^*T*^ with low 16S rRNA gene sequence similarity value of 96.3%. Strain CMU55-4 is closely related to *Micromonospora chalcea* DSM 43026^*T*^ with 16S rRNA gene sequence similarity value of 98.2%. The ANIb values of 98.47% and digital DNA-DNA hybridization value of 91.8% were found between strain CMU55-4 and *M. chalcea* DSM 43026^*T*^.

**TABLE 2:** Taxonomic assignment of bryophytes associated actinobacteria based on EzBiocloud database.

**Bacterial strain**	**Isolation source**	**Media**	**DDBJ accession number**	**Different nt/Total nt**	**Similarity (%)**	**Top hit taxon**
CMU15-1	*B. apiculatum*	Water proline agar	LC458843	5/1,373	99.63	*Micromonospora humi* DSM 45647^*T*^
CMU15-2	*B. apiculatum*	Water proline agar	LC458844	7/1,343	99.48	*Micromonospora humi* DSM 45647^*T*^
CMU15-3	*B. apiculatum*	Water proline agar	LC458845	11/1,362	99.19	*Micromonospora humi* DSM 45647^*T*^
CMU15-4	*B. apiculatum*	Water proline agar	LC458846	11/1,366	99.19	*Micromonospora citrea* DSM 43903^*T*^
CMU51-1	*S. gemmascens*	Starch casein agar	LC458847	0/1,357	100	*Streptomyces fulvissimus* DSM 40593^*T*^
CMU51-2	*S. gemmascens*	Starch casein agar	LC458848	2/1,395	99.85	*Streptomyces pratensis* ch24^*T*^
CMU51-4	*S. gemmascens*	Water proline agar	LC458849	3/1,370	99.78	*Streptomyces pratensis* ch24^*T*^
CMU51-5	*S. gemmascens*	R2A agar	LC458850	2/1,366	99.85	*Streptomyces fulvissimus* DSM 40593^*T*^
CMU55-1	*C. involutus*	Humic acid vitamin B agar	LC458851	2/1,366	99.85	*Micromonospora tulbaghiae* DSM 45142^*T*^
CMU55-2	*C. involutus*	Water proline agar	LC458852	0/1,339	100	*Mycolicibacterium iranicum* DSM 45541^*T*^
CMU55-3	*C. involutus*	Humic acid vitamin B agar	LC458853	6/1,376	99.56	*Streptomyces fulvissimus* DSM 40593^*T*^
CMU55-4	*C. involutus*	Water proline agar	LC458854	22/1,342	98.20	*Micromonospora chalcea* DSM 43026^*T*^
CMU55-5	*C. involutus*	Humic acid vitamin B agar	LC438389	53/1,544	96.31	*Microbispora rosea* subsp. *rosea* ATCC 12950^*T*^

### Growth Promotion on *Physcomitrium sphaericum* (C. Ludw.) Fürnr.

**FIGURE 2:**
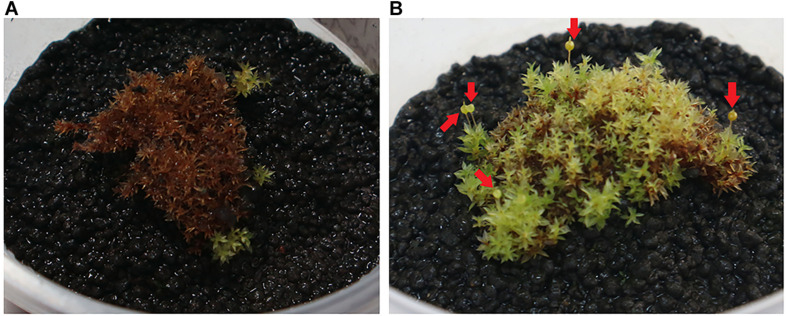
*P. sphaericum* control plant **(A)** and *P. sphaericum* inoculated with CMU55-4 for 1 month **(B)**. Arrows indicated capsule production.

**FIGURE 3:**
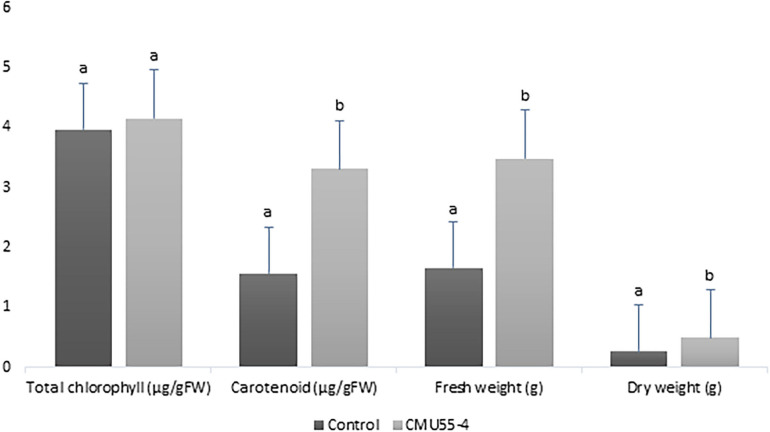
Effects of *Micromonospora* strain CMU55-4 toward the growth of *P. sphaericum.* a, b indicated significant difference in statistical analysis tested by SPSS independent *T*-test (*p* < 0.05), *n* = 3.

*Physcomitrium sphaericum* (C. Ludw.) Fürnr. plantlets that were inoculated with *Micromonospora* strain CMU55-4 showed better growth appearance compared to control plants. The control plants have more chlorosis leaves and showed growth retardation (Figure 2A). The plants that had been treated with strain CMU55-4 started to develop new leaves and showed the production of capsules (Figure 2B). Carotenoid, fresh weight and dry weight of *P. sphaericum* were significantly higher than the control. However, total chlorophyll content was not different in control and strain CMU55-4 inoculated plantlets (Figure 3).

### Genome Mining for Plant Growth Promoting Potential

**FIGURE 4:**
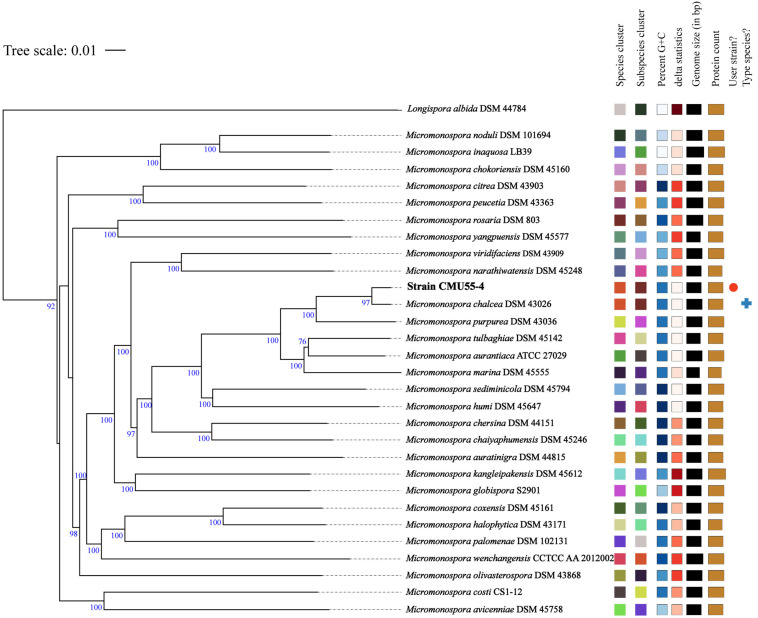
Phylogenomic tree of strain CMU55-4 and their closely related type strains of the genus *Micromonospora*. Tree inferred with FastME 2.1.6.1 (Lefort et al., 2015) from Genome BLAST Distance Phylogeny (GBDP) distances calculated from genome sequences. The branch lengths are scaled in terms of GBDP distance formula d_5_. The numbers above branches are GBDP pseudo-bootstrap support values from 100 replications, with an average branch support of 92.6 %. The tree was rooted at the midpoint (Farris, 1972). Leaf labels were annotated by affiliation to species and subspecies clusters, genomic G+C content, δ values, overall genome sequence length, number of proteins, and the kind of strain, respectively.

From whole genome sequence data, the estimated genome size of *Micromonospora* sp. CMU55-4 was 6.6 Mb. The GC content was 73.0%. The summary of sequence assembly and genome annotation is displayed in Table 3. Phylogenomic tree clearly classified isolate CMU55-4 in the same species and subspecies level with *M. chalcea* DSM 43026^*T*^ (Figure 4). Prokka predicted 5,946 coding regions with 6,077 estimated genes (Table 4) contained 67 tRNAs, 3 rRNAs and 2 repeat regions. Gene annotation in RAST server showed that genes were grouped into 25 subsystems (Supplementary Material 1). The majority of genes play roles in amino acids, carbohydrate, and protein metabolisms. From various subsystems, essential roles of protein coding sequences according to their plant growth promoting traits was grouped in Table 5. Twenty-four genes were associated with siderophore production, namely siderophore assembly kit, siderophore desferrioxamine E, and siderophore aerobactin. Indole-3-glycerol phosphate synthase and tryptophan synthase plays role in IAA synthesis. Gene encoding exopolyphosphatase with possible role in phosphate solubilization was also found. In addition, protein coding sequences involved in nitrogen metabolism such as assimilatory nitrate reductase, nitrate/nitrite transporter, and ammonium transporter were also detected. Strain CMU55-4 was well equipped with protein coding sequences related to oxidative stress response such as SoxR, NsrR, organic hydroperoxide reductase, glutathione peroxidases and trehalose synthesis genes. Glycine betaine transporter (OpuD) for osmotic adjustment under osmotic stress was also present. CspA protein family, DnaK and DnaJ chaperones responsible for cold or heat stress were identified. Strain CMU55-4 also exhibited genes involved in the utilization of saccharides found inside plant cells such as xylose, arabinose, mannose and D-galacturonate (Table 5).

**TABLE 3:** Summary of sequence reads assembly of strain CMU55-4.

	**CMU55-4**
Total number of raw reads	1,878,055
Percentage of bases > Q30	75
Number of contigs	68
Largest contig	661,139
GC (%)	72.98
N50	319,632
L50	7
Average coverage	120
Total bases	6,624,976

**TABLE 4:** Summary of genome annotation of strain CMU55-4.

	**CMU55-4**
**Prokka**	
Number of predicted rRNA	3
Number of predicted CDS	5,946
Number of predicted tRNA	67
Number of predicted tmRNA	1
Number of predicted misc_RNA	60
Number of repeat region	2
Number of predicted gene	6,077
**NCBI PGAP**	
Genes (total)	6,056
CDSs (total)	5,999
Genes (coding)	5,910
CDSs (with protein)	5,910
Genes (RNA)	57
rRNAs	1, 1, 1 (5S, 16S, 23S)
complete rRNAs	1, 1, 1 (5S, 16S, 23S)
tRNAs	51
ncRNAs	3
Pseudo Genes (total)	89
CDSs (without protein)	89
Pseudo Genes (ambiguous residues)	0 of 89
Pseudo Genes (frameshifted)	18 of 89
Pseudo Genes (incomplete)	74 of 89
Pseudo Genes (internal stop)	7 of 89
Pseudo Genes (multiple problems)	8 of 89
CRISPR Arrays	2

**TABLE 5:** Protein coding sequences related with plant growth promoting traits of *M. chalcea* CMU55-4.

**PGP traits**	**Protein coding sequences conferring PGP traits**
IAA	Indole-3-glycerol phosphate synthase (EC 4.1.1.48)
	Tryptophan synthase alpha chain (EC 4.2.1.20)
	Tryptophan synthase beta chain (EC 4.2.1.20)
Siderophore	*Siderophore assembly kit*:
production	1. Isochorismatase (EC 3.3.2.1) of siderophore biosynthesis
	2. Siderophore biosynthesis L-2,4-diaminobutyrate decarboxylase
	3. Siderophore synthetase component, ligase
	4. Ferric hydroxamate ABC transporter (TC 3.A.1.14.3), permease component FhuB
	5. ABC-type Fe^3+^-siderophore transport system, ATPase component
	6. ABC-type Fe^3+^-siderophore transport system, permease 2 component
	7. Thioesterase in siderophore biosynthesis gene cluster
	8. Siderophore synthetase small component, acetyltransferase
	9. ABC-type Fe^3+^-siderophore transport system, permease component
	10. Siderophore biosynthesis protein, monooxygenase
	11. Putative ABC iron siderophore transporter, fused permease and ATPase domains
	12. 2,3-dihydro-2,3-dihydroxybenzoate dehydrogenase (EC
	1.3.1.28) of siderophore biosynthesis
	13. Ferric hydroxamate ABC transporter (TC 3.A.1.14.3), ATP-binding protein FhuC
	14. Ferric hydroxamate ABC transporter (TC 3.A.1.14.3), periplasmic substrate binding protein FhuD
	15. Thioesterase in siderophore biosynthesis gene cluster
	16. Siderophore synthetase small component, acetyltransferase
	17. ABC-type Fe^3+^-siderophore transport system, permease component
	18. Siderophore biosynthesis protein, monooxygenase
	19. Putative ABC iron siderophore transporter, fused permease and ATPase domains
	20. 2,3-dihydro-2,3-dihydroxybenzoate dehydrogenase (EC 1.3.1.28) of siderophore biosynthesis
	21. Ferric hydroxamate ABC transporter (TC 3.A.1.14.3), ATP-binding protein FhuC
	21. Ferric hydroxamate ABC transporter (TC 3.A.1.14.3),
	periplasmic substrate binding protein FhuD
	*Siderophore Desferrioxamine E*:
	1. Desferrioxamine E biosynthesis protein DesA, DesB, DesC, DesD
	*Siderophore Aerobactin:*
	1. Ferric hydroxamate ABC transporter (TC 3.A.1.14.3), permease component FhuB
	2. Ferric hydroxamate ABC transporter (TC 3.A.1.14.3), ATP-binding protein FhuC
	2. Ferric hydroxamate ABC transporter (TC 3.A.1.14.3), periplasmic substrate binding protein FhuD
Nitrogen	*Nitrate and nitrite ammonification*:
metabolism	1. Assimilatory nitrate reductase large subunit (EC 1.7.99.4)
	2. Nitrate/nitrite transporter
	3. Nitrite reductase [NAD(P)H] small subunit (EC 1.7.1.4)
	4. Nitrite reductase [NAD(P)H] large subunit (EC 1.7.1.4)
	*Ammonia assimilation*:
	1. Ferredoxin-dependent glutamate synthase (EC 1.4.7.1)
	2. Nitrogen regulatory protein P-II
	3. Glutamate-ammonia-ligase adenylyltransferase (EC 2.7.7.42)
	4. Ammonium transporter
	5. Glutamate synthase [NADPH] large chain (EC 1.4.1.13)
	6. Glutamine synthetase type I (EC 6.3.1.2)
	7. [Protein-PII] uridylyltransferase (EC 2.7.7.59)
	8. Glutamate synthase [NADPH] small chain (EC 1.4.1.13)
Phosphate solubilization	Exopolyphosphatase (EC 3.6.1.11)
Osmotic stress alleviation	Glycine betaine transporter OpuD
Oxidative stress response	1. Redox-sensitive transcriptional activator SoxR
	2. Nitrite-sensitive transcriptional repressor NsrR
	3. Organic hydroperoxide resistance transcriptional regulator
	4. Transcriptional regulator, Crp/Fnr family
	5. Alkyl hydroperoxide reductase subunit C-like protein
	6. Phytochrome, two-component sensor histidine kinase (EC 2.7.3.-)
	7. Organic hydroperoxide resistance protein
	Glutathione: Biosynthesis and gamma-glutamyl cycle
	1. Gamma-glutamyltranspeptidase (EC 2.3.2.2)
	2. Glutamate-cysteine ligase (EC 6.3.2.2)
	Glutathione: Non-redox reactions
	1. Lactoylglutathione lyase (EC 4.4.1.5), Glutathione S-transferase, omega (EC 2.5.1.18)
	2. CoA disulfide thiol-disulfide redox system = CoA-disulfide reductase (EC 1.8.1.14)
	Glutathione: Redox cycle
	1. Glutathione peroxidase (EC 1.11.1.9)
Cold shock protein	Cold shock, CspA family of proteins:
	1. Cold shock protein CspA
	2. Cold shock protein CspC
Heat shock protein	Heat shock dnaK gene cluster extended:
	1. Hypothetical radical SAM family enzyme in heat shock gene cluster, similarity with CPO of BS HemN-type
	2. HspR, transcriptional repressor of DnaK operon
	3. Heat-inducible transcription repressor HrcA
	4. Chaperone protein DnaK
	5. Chaperone protein DnaJ
	6. Ribosomal RNA small subunit methyltransferase E (EC 2.1.1.-)
	7. tmRNA-binding protein SmpB
	8. Heat shock protein GrpE
	9. Translation elongation factor LepA
	10. Nucleoside 5-triphosphatase RdgB (dHAPTP, dITP, XTP-specific) (EC 3.6.1.15)
	11. Ribonuclease PH (EC 2.7.7.56)
	12. Signal peptidase-like protein
	13. rRNA small subunit methyltransferase I
Chitinase production	Chitinase (EC 3.2.1.14)
Carotenoid production	Zeaxanthin glucosyl transferase
Trehalose	1. Trehalose synthase (EC 5.4.99.16)
metabolism	2. Malto-oligosyltrehalose synthase (EC 5.4.99.15)
	3. 1,4-alpha-glucan (glycogen) branching enzyme, GH-13-type (EC 2.4.1.18)
	4. Trehalose-6-phosphate phosphatase (EC 3.1.3.12)
	5. Putative glucanase *glg*E (EC 3.2.1.-)
	6. Malto-oligosyltrehalose trehalohydrolase (EC 3.2.1.141)
	7. Glucoamylase (EC 3.2.1.3)
Utilization of	*Xylose utilization*:
sugar found	1. Xylose isomerase (EC 5.3.1.5)
inside plant	2. Xylulose kinase (EC 2.7.1.17)
cells	3. Endo-1,4-beta-xylanase A precursor (EC 3.2.1.8)
	4. Xylose-responsive transcription regulator, ROK family
	*Arabinose utilization*:
	1. L-ribulose-5-phosphate 4-epimerase (EC 5.1.3.4)
	2. Ribulokinase (EC 2.7.1.16)
	3. L-arabinose isomerase (EC 5.3.1.4)
	4. Arabinan endo-1,5-alpha-L-arabinosidase (EC 3.2.1.99)
	*D-Galacturonate and D-Glucuronate Utilization*:
	1. Mannonate dehydratase (EC 4.2.1.8)
	2. D-mannonate oxidoreductase (EC 1.1.1.57)
	3. Endo-1,4-beta-xylanase A precursor (EC 3.2.1.8)
	4. Uronate isomerase (EC 5.3.1.12)
	5. Alpha-glucosidase (EC 3.2.1.20)
	6. Pectate lyase precursor (EC 4.2.2.2)
	7. 2-dehydro-3-deoxyphosphogluconate aldolase (EC 4.1.2.14)
	*Mannose metabolism*:
	1. Mannose-6-phosphate isomerase (EC 5.3.1.8)
	2. Beta-mannosidase (EC 3.2.1.25)
	D-ribose utilization
	1. Ribokinase (EC 2.7.1.15)
	Glucose and fructose metabolism:
	Genes in glycolysis and gluconeogenesis:
	1. Fructose-1,6-bisphosphatase, GlpX type (EC 3.1.3.11)
	2. Fructose-bisphosphate aldolase class II (EC 4.1.2.13)
	3. Pyrophosphate-dependent fructose 6-phosphate-1-kinase (EC 2.7.1.90)
	4. Glucose-6-phosphate isomerase (EC 5.3.1.9)
	5. Polyphosphate glucokinase (EC 2.7.1.63)
	6. Glucokinase (EC 2.7.1.2)
	*Genes in PPP pathway*:
	1. Glucose-6-phosphate 1-dehydrogenase (EC 1.1.1.49)
	2. Transketolase, N-terminal section (EC 2.2.1.1)
	3. Ribose-phosphate pyrophosphokinase (EC 2.7.6.1)
	4. Transketolase, C-terminal section (EC 2.2.1.1)
	5. Ribulose-phosphate 3-epimerase (EC 5.1.3.1)
	6. Glucose-6-phosphate 1-dehydrogenase (EC 1.1.1.49)
	7. 6-phosphogluconate dehydrogenase, decarboxylating (EC 1.1.1.44)
	8. 6-phosphogluconolactonase (EC 3.1.1.31), eukaryotic type
	9. Xylulose-5-phosphate phosphoketolase (EC 4.1.2.9)
	10. Fructose-6-phosphate phosphoketolase (EC 4.1.2.22)
	11. Transaldolase (EC 2.2.1.2)

### Chemotaxonomic, Cultural, and Phenotypic Characterizations of *Micromonospora* sp. CMU55-4

**FIGURE 5:**
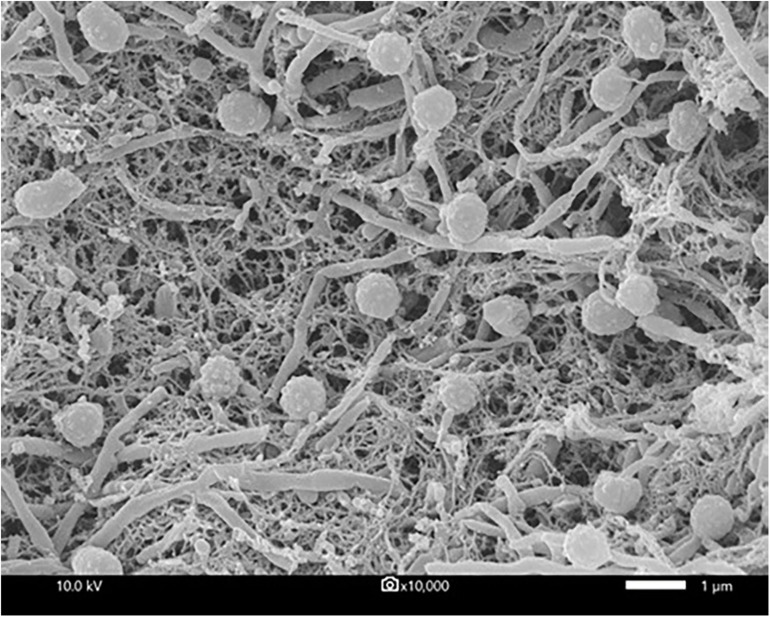
Scanning electron micrograph showing *M. chalcea* CMU55-4 grown on ISP2 agar medium at 30°C for 21 days. Bar represents 1 μm.

Strain CMU55-4 grew well on TSA, modified Bennett’s, and ATCC172 media. The colors of substrate mycelia were strong orange on ISP2, deep yellowish-brown on ISP1 and ISP3, ISP5, and ISP7, strong orange yellow on ISP4, ISP6 and TSA. Dark olive brown on modified Bennett’s and vivid orange on ATCC172. No growth was observed on NA and PDA. The strain produced globular spores (0.8 μm in size) with warty surface (Figure 5). The strain grew at 20 to 45°C (optimally at 30°C), pH 5.0 to 12.0 (optimally pH at 8.0) and tolerated up to 4% (w/v) NaCl. Strain CMU55-4 utilized various type of carbohydrates such as L-arabinose, dulcitol, D-mannose, and D-mannitol and those found in plant cells including D-glucose, sucrose, cellulose, amygdalin, and starch (Table 6). Cell-wall peptidoglycan of strain CMU55-4 contained *meso*-diaminopimelic acid. Galactose, arabinose and xylose were detected as diagnostic sugars in the whole-cell hydrolysates. The predominant phospholipids were diphosphatidylglycerol (DPG), phosphatidylethanolamine (PE), and phosphatidylinositol (PI). Major menaquinones were MK-9(H_4_) (33.46%), MK-9(H_6_) (13.63%), MK-9(H_8_) (26.16%), and MK-10(H_8_) (15.38%).

**TABLE 6:** Differential phenotypic characteristics of strain CMU55-4 and its phylogenetically closest type strains.

**Characteristics**	**1**	**2**	**3**
Peptonization	+	+	–
Coagulation	+	+	–
Nitrate reduction	–	–	+
Growth at 45°C	+	–	–
Growth at pH 12.0	+	+	–
Growth at 4% NaCl	+	–	–
Utilization of:			
L-Arabinose	+	+	–
Glycerol	–	+	+
Dulcitol	+	+	–
D-mannose	+	–	–
D-mannitol	+	+	–
D-Raffinose	–	+	+
D-Rhamnose	–	–	+
API ZYM			
Acid phosphatase	+	w	+
Alkaline phosphatase	+	w	+
α-Chymotrypsin	–	–	+
α-Galactosidase	+	w	+
β-Galactosidase	+	w	+
Lipase (C14)	–	w	–
Naphthol-AS-BI-phosphohydrolase	w	w	+
Trypsin	w	+	+
Valine arylamidase	+	w	+

**Strains: 1, CMU55-4; 2, M. chalcea DSM 43026^*T*^; 3, M. terminaliae TMS7^*T*^. +, Positive; −, negative; w, weakly positive; All phenotypic data were obtained from this study.**

## Discussion

Actinobacteria from bryophytes are very rare. In the earliest report in 2007, small number of *Rothia*, *Arthrobacter*, *Micrococcus*, and *Plantibacter* isolates were reported as endophytic actinobacteria from *Sphagnum magellanicum* Brid. and *Sphagnum fallax* H. Klinggr (Opelt et al., 2007). Only three new species of actinobacteria were described from unidentified moss species namely *Streptomyces bryophytorum* (Li et al., 2016), *Actinoallomulus bryophytorum* (Li et al., 2015a) and *Microbispora bryophytorum* (Li et al., 2016). In this study, almost half of actinobacteria (46%) were identified as *Micromonospora*. Members of this genus have been isolated from various habitats including plants (Trujillo et al., 2010, 2015; Carro et al., 2012, 2013; Genilloud, 2015). Several new species of *Micromonospora* are endophytes of various plants as exemplified by the description of *M. costi* (Thawai, 2015), *M. globbae* (Kuncharoen et al., 2018), *M. phytophila* (Carro et al., 2018b), *M. luteiviridis* (Carro et al., 2018b), *M. oryzae* (Kittiwongwattana et al., 2015), and *M. terminaliae* (Kaewkla et al., 2017). *Streptomyces* isolates also dominant represent around 38.5%. Our results provide additional evidence that these two actinobacterial genera are also dominant in bryophytes. *Mycolicibacterium stellerae* was recently described as endophytic actinobacteria from plant, *Stellera chamaejasme* L. in Yunnan (Nouioui et al., 2019b). However, this is the first time that member of this genus was found associated with a bryophyte. The remaining isolate, CMU55-5 was closely related to *Microbispora rosea* subsp. *rosea*. The low 16S rRNA gene similarity value (96.3%) between CMU55-5 and its closest neighbor, suggests that this strain may represent a new species. However, polyphasic taxonomic characterization is needed for its formal description which will be the subject of future investigation.

No actinobacteria was obtained from liverwort. This may be an effect of “oil bodies,” the highly distinctive organelle uniquely found in liverworts (He et al., 2013). This organelle is responsible for toxic compound accumulation that usually use for protection of liverworts from herbivore (Stahl, 1888), pathogens, low temperature and excessive light (Hieronymus, 1892). We opined that during the isolation of actinobacteria, the liverwort sample was crushed which may cause the release of toxic compounds from oil bodies and inhibit the growth of actinobacteria.

The use of water proline agar yielded the highest number of actinobacterial isolates in this study. Water proline agar is a low nutritional medium that simulate oligotrophic status in natural environments. Proline is also serving as a compatible solute that commonly found in plant cells. The composition of the minimal media is suggested to allow an easier adaptation for endophytes (Alain and Querllou, 2009) and allow slow growing endophytic strains a chance to develop (Eevers et al., 2015). Complex media composed of rich carbon and nitrogen sources are suggested to be unsuitable for the growth of slow-growing endophytic bacteria as they do not resemble the environment inside plant tissues (Eevers et al., 2015). Low nutritional media have been successfully used to isolate actinobacteria from various environmental samples (Janssen et al., 2002; Wang D.-S. et al., 2014; Ruttanasutja and Pathom-aree, 2015).

Strain CMU55-4 was classified into the genus *Micromonospora* of the family *Micromonosporaceae* based on the sequence of 16S rRNA gene and its unique phenotypic characteristics. It forms a characteristic orange colony at the early stage, which turns to dark olive brown color with age. The strain produced extensively branched substrate hyphae, lack of aerial mycelia which is a unique morphological characteristic of the genus *Micromonospora* (Ørskov, 1923). The strain formed a single non-motile warty-globular spore on the vegetative mycelium similar to the closest type strains, *M. chalcea* DSM 43026^*T*^ (Ørskov, 1923). The formation of single *spores* is also the main *morphological* characteristic of the genus *Micromonospora (Trujillo et al., 2015)*. A *meso*-diaminopimelic acid was found in the cell wall peptidoglycan of strain CMU55-4 with arabinose, xylose and galactose as diagnostic sugars in the whole-cell hydrolysates corresponding to the cell wall type II and sugar type D (Lechevalier et al., 1971). Strain CMU55-4 exhibited phospholipid type II, comprising of phosphatidylethanolamine (PE), diphosphatidylglycerol (DPG), and phosphatidylinositol (PI) as the major phospholipids (Lechevalier et al., 1977). The strain contained a large amount of MK-9(H_4_) and MK-9(H_6_) which generally found in the genus *Micromonospora* (Nouioui et al., 2018). Strain CMU55-4 shared 98.47% of ANIb values and 91.8% digital DNA-DNA hybridization to *M. chalcea* DSM 43026^*T*^. The value of <95–96% ANI or 70% dDDH is proposed as a cutoff value for delineating of new species (Chun et al., 2018). Therefore, strain CMU55-4 was identified as *M. chalcea* CMU55-4.

*Micromonospora* have long been recognized as antibiotic producers (Hirsch and Valdes, 2010). Recently, endophytic *Micromonospora* have been regarded as plant growth promoting bacteria especially in legumes (Trujillo et al., 2014; Benito et al., 2017). *Physcomitrium sphaericum* moss is known to be distributed only in the temperate regions; North America, Europe, Russia, China, and Japan. Recently, it has been found in the tropical country and reported as new record to Thailand (Printarakul et al., 2014). The inoculation of *M. chalcea* CMU55-4 on *P. sphaericum* moss resulted in an increase of carotenoid content, fresh and dry weight significantly higher than the control (Figure 3). This promotion effect appears to be related to the production of phytohormone IAA as *M. chalcea* CMU55-4 produced the highest IAA among all tested actinobacteria. Interestingly, *P. sphaericum* inoculated with *M. chalcea* CMU55-4 developed new leaves and showed the production of capsules (Figure 2B). Capsule is an important part of bryophyte reproduction as it represent sporophyte stage and benefits in asexual reproduction through spores dispersion (Sundberg et al., 2014). It is likely that the moss-bacterium interaction between *M. chalcea* CMU55-4 and *P. sphaericum* may play a role in promote leaves and capsule development though the exact mechanism still required further investigation. Effect of moss associated bacteria on *Pylaisiella selwynii* moss development was reported that the obtained Gram-negative bacteria could promote protonemal growth and gametophore initiation of *P. selwynii* (Spiess et al., 1981). Recently, auxin has been shown to involve in the development of moss (*Physcomitrella patens*) including gametophore and sporophyte development (Thelander et al., 2018). Almost all works regarding plant growth promoting actinobacteria are carried out with vascular plants. For examples, the inoculation of *Micromonospora* strain SB3 promotes plant biomass, root length and increases the acclimatization rate of *Lolium multiflorum* plantlets after 4 weeks of *in vitro* culture (Della Mónica et al., 2018). Recently, an endophytic *M. chalcea* UAE1 from halophytic crop, *Salicornia bigelovii* has been reported to promote the growth of *S. bigelovii* mainly by ACC-deaminase production (El-Tarabily et al., 2019). To the best of our knowledge, this is the first report on potential of *Micromonospora* species on the growth and development of non-vascular plants such as mosses.

Approximate genome sizes of 40 *Micromonospora* type strains were reported to be ranged from 6.1 Mbp for *Micromonospora marina* DSM 45555^*T*^ to 7.9 Mbp for *M. carbonacea* DSM 43168^*T*^ with the average genome size for all of the *Micromonospora* strains was 7 ± 0.4 Mbp (Carro et al., 2018a). The estimated genome size of our *M. chalcea* CMU55-4 was within these previously reported ranges (6.6 Mb) with 73.0% GC content. The genome size of *M. chalcea* DSM 43026^*T*^ was 7.0 Mb and 72.8% G+C content (Carro et al., 2018a). The slightly bigger genome size of 7.086 Mb was reported from plant growth promoting rhizosphere *Micromonospora* sp. strain MW-13 with a similar G+C content of 73.3% (Jahanshah et al., 2019).

Omics techniques, including genome sequencing, comparative genomics, microarray, next generation sequencing, metagenomics, and metatranscriptomics can be used to explain plant-endophyte relationship (Kaul et al., 2016). Endophytic lifestyle of *M. chalcea* CMU55-4 was supported by data from genome mining and phenotypic studies as this strain was able to utilize various kind of saccharides and carbohydrates which found inside plant cells. Saccharides molecules were discharged from plant cell walls during elongation process and served as nutrients to promote the colonization ability of plant-associated bacteria (Etesami et al., 2015). *M. chalcea* CMU55-4 is also able to produce both catecholate and hydroxamate type siderophores *in vitro*. In addition, several predictive genes related to siderophore production were also detected in its genome for examples desferrioxamine E and aerobactin and genes encoding transporter proteins. Neilands and Leong (1986) suggested that siderophore systems may play a role in infections of plant tissue. Thus, the ability of *M. chalcea* CMU55-4 to produce siderophores may help this strain to enter and live inside plant cells. Metallophores producing *Micromonospora* strains were recently reported to promote the growth of *Arabidopsis thaliana* under heavy metal condition by playing roles in metal acquisition, iron metabolism and resistance to toxic compounds (Ortúzar et al., 2020). Genes mutually found in Ortúzar et al. (2020) and this study are ferric hydroxamate ABC transporter (FhuB, FhuC, FhuD), siderophore desferrioxamine E (DesA, DesB, DesC, DesD), and siderophore aerobactin (FhuB, FhuC, FhuD).

Previous studies on actinobacterial genome revealed genes related to plant growth promoting activities primarily production of IAA, siderophore, phosphate solubilization, and phytopathogen inhibition (Gupta et al., 2014; Liu et al., 2019; Nouioui et al., 2019a). Predictive genes obtained from genome mining in this study supported the experimental data on IAA and siderophore producing abilities of *M. chalcea* CMU55-4 *in vitro*. Tryp-dependent pathway could be a pathway for IAA synthesis in strain CMU55-4 as predictive genes for tryptophan synthase alpha chain (TSA1), tryptophan synthase beta chain (TSB), indole-3-glycerol phosphate synthase (IGS) were found. Indole-3-glycerol phosphate or indole is a branch point for Tryp-dependent pathway that directly leads to IAA synthesis (Spaepen and Vanderleyden, 2011; Di et al., 2016). IAA promotes plant growth by advocating cell division, cell elongation, stimulates primary growth, and plays role in stress resistant. IAA production trait is considered as a useful criterion for selection of endophytic and rhizospheric bacteria in rice growth promotion (Etesami et al., 2015). High IAA production without L-tryptophan was recorded in some isolates such as CMU51-1 and CMU51-5. This observation suggests the possibility that these isolates can produce IAA by Tryp-independent pathway. *M. chalcea* CMU55-4 failed to solubilize tricalcium phosphate on PVK agar though predictive gene for exopolyphosphatase was detected. Since only tricalcium phosphate was used for phosphate solubilizing property, we cannot rule out the possibility of *M. chalcea* CMU55-4 to solubilize other types of inorganic phosphates. *M. chalcea* CMU55-4 also genetically possessed abilities related to nitrogen metabolism in particular nitrate/nitrite ammonification and ammonia assimilation. These abilities could enhance nitrogen uptake system of plant as nitrate (NO_3_^–^) and ammonium (NH_4_^+^) are the main resources of inorganic nitrogen (N_2_) absorbed by the roots of higher plants (Wickert et al., 2007).

It is also interesting to note the gene sequences responsible for other functions, such as the production of antioxidants and heat shock/cold shock proteins were detected though these properties had not been investigated under laboratory conditions in this study. *M. chalcea* CMU55-4 contained predictive genes involved with oxidative stress response such as SoxR, NsrR, organic hydroperoxide resistance protein, trehalose synthesis genes, glutathione S-transferase omega, and carotenoid production, which are the molecules help plant to tolerate to oxidative stress. Reactive oxygen species (ROS) created by the plant cells, are generally neutralized by the production of enzymes such as superoxide dismutases (SOD), catalases (CatA), peroxidases (POD), alkyl hydroperoxide reductases (AhpC), genes conferring nitrosative stress caused by reactive nitrogen species, and glutathione-S-transferases (GSTs) in endophytes (Khare et al., 2018). GST was found in the genome of *Streptomyces scabrisporus* NF3, an endophyte of *Amphipterygium adstringens* (Ceapã et al., 2018). The ability to alleviate oxidative stress is one attractive property of plant growth promoting bacteria. Forty-two genomes of *Micromonospora* strains including the type strain of *M. chalcea*, were analyzed to determine the presence of PGP genes and other characteristics related with plant growth promotion (Carro et al., 2018a). Genomes of *Micromonospora* strains contain genes related to stress responses such as *csp*A, *csp*C for cold shock response, *dna*K and *grp*E for heat shock responses, and *sod* for oxidative stress. Similar observation was also found in this study.

Bryophytes require high water for growth. They are more sensitive to water deficit condition than other plants. Accumulation of compatible solute such as glycine betaine therefore can help bryophytes to maintain water balance. Glycine betaine transporter OpuD was found in *M. chalcea* CMU55-4 genome. The opuD gene product was reported to be essential for glycine betaine uptake and osmoprotection in *E. coli* (Kappes et al., 1996). Glycine betaine accumulation had been reported to increase the drought tolerant in *Streptomyces chatreusis* (Wang et al., 2019). Predictive genes involve in trehalose metabolism was also present in *M. chalcea* CMU55-4 genome including gene encoded for trehalose synthase. Trehalose is another compatible solute that can protect plant proteins and cellular membranes from inactivation or denaturation caused by a variety of stress conditions, including desiccation, dehydration, heat, cold, and oxidation (Elbein et al., 2003).

Bryophytes usually grow in low temperature and high humidity area. Cold shock protein may be essential for the adaptation of actinobacteria to live with bryophytes under such environment. *M. chalcea* CMU55-4 genome is equipped with CspA family of cold shock protein and several heat shock protein genes. This protein plays an essential role in inhibition of DNA replication during cold-adaptation in *Streptomyces* sp. AA8321 (Kim et al., 2007). Similarly, heat shock proteins (HSP) can be benefit to bryophytes for survival under high temperature. HSPs remove denatured proteins to prevent formation of large protein aggregates and cell death while DnaK/DnaJ/GrpE or GroEL/GroES chaperones are part of sigma-32 heat shock regulon that regulates cell during heat stress and maintains protein homeostasis in living cells (Schumann, 2016).

In conclusion, this study provides the first evidence of cultivable actinobacteria associated with three high altitude moss species, *Bryum apiculatum*, *Syntrichia gemmascens* and *Campylopus involutus*. These actinobacteria show plant growth promoting ability *in vitro*. The inoculation of the selected strain, *M. chalcea* CMU55-4, can promote the growth of rare moss species, *P. sphaericum*. The response of this moss to *M. chalcea* CMU55-4 suggested that actinobacteria of the genus *Micromonospora* might occur naturally in association with the moss and might commonly affect moss development in nature. Genome mining data also support plant growth promotion potential of *M. chalcea* CMU55-4 as a good candidate for breeding program of *P. sphaericum* especially those under acclimatization and other moss species.

## Data Availability Statement

The datasets generated for this study can be found in online the repositories. The names of the repository/repositories and accession number(s) can be found below: https://www.ncbi.nlm.nih.gov/genbank/, JAAOLH000000000.

## Author Contributions

CI performed overall experiments and wrote the manuscript. WP, NC, and ST supervised CI. CI, WP, NK, and NC revised the manuscript. WP conceived the idea and designed research outline. All authors contributed to the article and approved the submitted version.

## Conflict of Interest

The authors declare that the research was conducted in the absence of any commercial or financial relationships that could be construed as a potential conflict of interest.

## Footnotes

1 https://www.ezbiocloud.net/
