# Supplementary figures and images for: Bryophytes Harbor Cultivable Actinobacteria With Plant Growth Promoting Potential
Source: Front Microbiol. 2020 Sep 29;11:563047. doi: 10.3389/fmicb.2020.563047 (PMC7550540; doi:10.3389/fmicb.2020.563047)

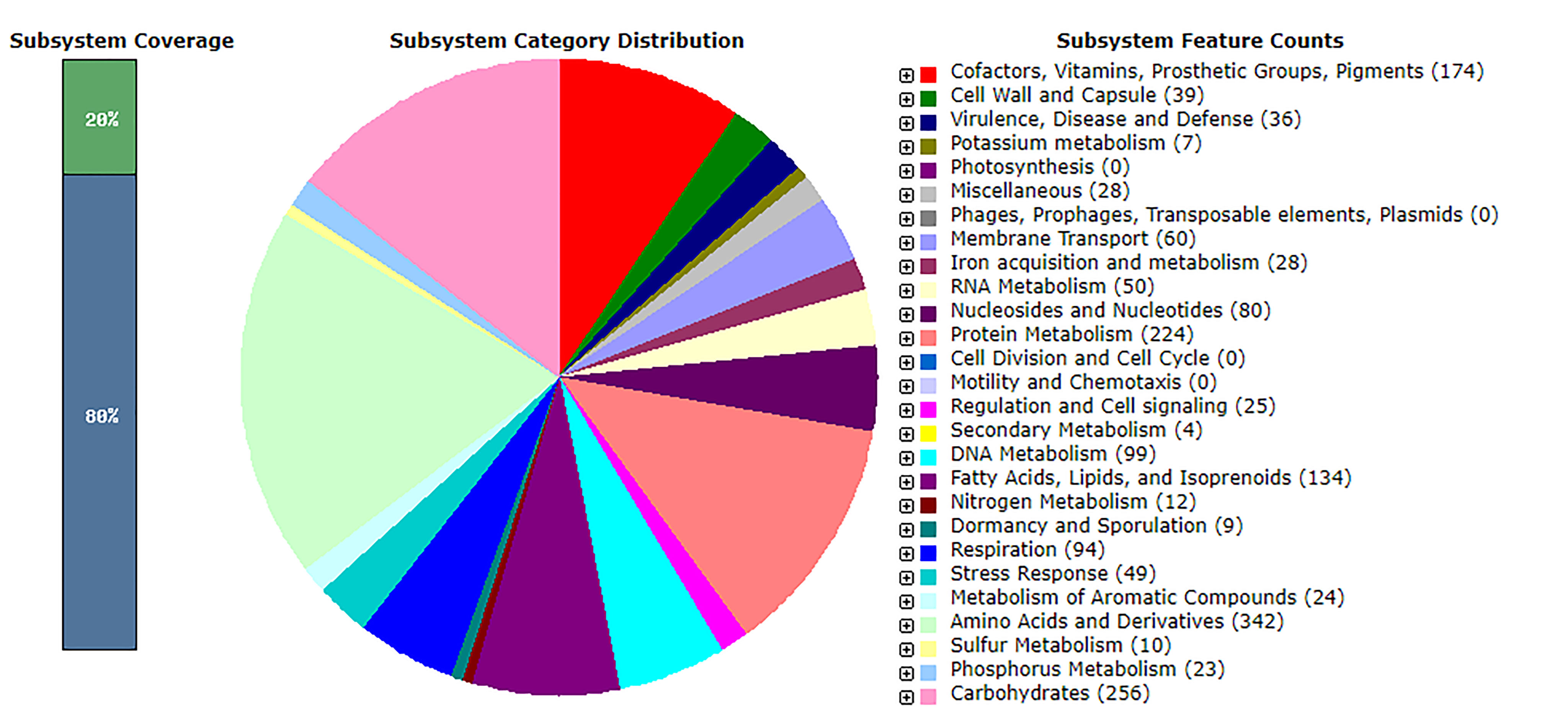

Supplement: Supplementary file 1 [file Image_1.JPEG]
